# Development of approaches to stratification of patients with schizophrenia based on cytokine levels using cluster analysis

**DOI:** 10.1192/j.eurpsy.2022.2007

**Published:** 2022-09-01

**Authors:** E. Ermakov, A. Boiko, D. Parshukova, E. Dmitrieva, S. Ivanova, V. Buneva

**Affiliations:** 1 Institute of Chemical Biology and Fundamental Medicine, Laboratory Of Repair Enzymes, Novosibirsk, Russian Federation; 2 Mental Health Research Institute, Tomsk National Research Medical Center of the Russian Academy of Sciences, Laboratory Of Molecular Genetics And Biochemistry, Tomsk, Russian Federation; 3 Tomsk National Research Medical Center of the Russian Academy of Sciences, Mental Health Research Institute, Tomsk, Russian Federation

**Keywords:** schizophrenia, cytokines

## Abstract

**Introduction:**

Alterations in a variety of immune parameters, including abnormal cytokine levels, are known to be found in schizophrenia. These changes can be useful in identifying patients with the most severe immune abnormalities.

**Objectives:**

To develop approaches to stratification of schizophrenia patients based on cytokine levels using cluster analysis.

**Methods:**

We recruited 53 patients (25 women/28 men) with a verified diagnosis of simple or paranoid schizophrenia and 37 healthy individuals (19 women/18 men) in our study. Serum levels of IL-1β, IL-2, IL-4, IL-6, TNFα, INFα, BAFF, GM-CSF, NGFβ, NRG1, and GDNF were determined using a MAGPIX multiplex analyzer (Luminex, USA). Statistical analysis was performed in Statistica 10.

**Results:**

Principal component analysis and partial least-squares discriminant analysis showed that the combined multi-cytokine profiles of the studied groups differ. The results of the k-means cluster analysis are presented in Table 1 The most reliable results are obtained by a combination of 4 variable: IL-1β, IL- 4, BAFF and GDNF. Table 1 Percent of individuals classified in different clusters depending of number of parameters using for classification.

**Table:** 

Number of variables for classification	Healthy individuals		Schizophrenia patients	
	Cluster 1	Cluster 2	Cluster 1	Cluster 2
10 variables	5,4	96,4	26,4	73,6
4 variables	0	100	11,1	88,9
3 variables	2,7	97,3	29,6	70,4
2 variables	8,1	91,9	20,4	79,6

**Conclusions:**

A subgroup (сluster 1) of schizophrenic patients with severe immune abnormalities was identified using data on the levels of IL-1β, IL-4, BAFF and GDNF. Anti-inflammatory therapy is recommended for this subgroup of patients. *Support by Grant of RSF № 21-75-00102.*

**Disclosure:**

No significant relationships.

